# Individual and community level socioeconomic inequalities in contraceptive use in 10 Newly Independent States: a multilevel cross-sectional analysis

**DOI:** 10.1186/1475-9276-11-69

**Published:** 2012-11-16

**Authors:** Teresa Janevic, Pallas W Sarah, Ismayilova Leyla, Bradley H Elizabeth

**Affiliations:** 1 Department of Epidemiology, UMDNJ School of Public Health, 683 Hoes Lane West, Piscataway, NJ, 08854, USA; 2 Department of Health Policy and Management, Yale School of Public Health, 60 College Street, New Haven, CT, 06520, USA; 3 School of Social Service Administration, University of Chicago, 969 East 60th Street, Chicago, IL, 60637, USA

## Abstract

**Introduction:**

Little is known regarding the association between socioeconomic factors and contraceptive use in the Newly Independent States (NIS), countries that have experienced profound changes in reproductive health services during the transition from socialism to a market economy.

**Methods:**

Using 2005–2006 data from Demographic Health Surveys (Armenia, Azerbaijan, and Moldova) and Multiple Indicator Cluster Surveys (Belarus, Georgia, Kazakhstan, Kyrgyzstan, Tajikistan, Ukraine, and Uzbekistan), we examined associations between individual and community socioeconomic status with current modern contraceptive use (MCU) among N = 55,204 women aged 15–49 married or in a union. Individual socioeconomic status was measured using quintiles of wealth index and education level (higher than secondary school, secondary school or less). Community socioeconomic status was measured as the percentage of households in the poorest quintile of the nationals household wealth index (0%, 0–25%, or greater than 25%). We used multilevel logistic regression to estimate associations adjusted for age, number of children, urban/rural, and socioeconomic variables.

**Results:**

MCU varied by country from 14% (in Azerbaijan) to 62% (in Belarus). Overall, women living in the poorest communities were less likely than those in the richest to use modern contraceptives (adjusted odds ratio (aOR) = 0.82, 95% Confidence Interval = 0.76, 0.89). Similarly, there was an increasing odds of MCU with increasing individual-level wealth. Women with a lower level of education also had lower odds of MCU than those with a higher level of education (aOR = .75, 95%CI = 0.71, 0.79). In country-specific analyses, community-level socioeconomic inequalities were apparent in 4 of 10 countries; in contrast, inequalities by individual-level wealth were apparent in 7 countries and by education in 8 countries. All countries in which community-level socioeconomic status was associated with MCU were in Central Asia, whereas at the individual-level inequalities of the largest magnitude were found in the Caucasus. There were no distinct patterns found in Eastern European countries.

**Conclusions:**

Community-level socioeconomic inequalities in MCU were most pronounced in Central Asian countries, whereas individual-level socioeconomic inequalities in MCU were most pronounced in the Caucasus. It is important to consider multilevel contextual determinants of modern contraceptive use in the development of reproductive health and family planning programs.

## Introduction

Countries of the former Soviet Union, now referred to as the Newly Independent States, suffered during the Soviet-era from lack of access to modern contraception, and as a result continue to have among the high abortion rates in the world [1]. One study found about half of pregnancies in Eastern Europe and the Former Soviet Union were unintended, and that the vast majority of these pregnancies ended in induced abortion [2]. Although this region achieved a low fertility rate in the 20th century, this achievement was due less to modern contraceptive use (MCU) than to high rates of abortion, the primary method of controlling fertility during the Soviet era [3].

The transition from socialist to market-based economies in the Newly Independent States has had a profound impact on health services in the region. The health system of the Soviet Union was characterized by centralization, overuse of physicians in delivery of care, weak preventive health care systems, and overall lack of family planning programs [4]. After the transition, Russia, Ukraine, Belarus, Azerbaijan, Tajikistan, Turkmenistan, and Uzbekistan retained most of the Soviet-era health system, whereas Moldova, Georgia, Armenia, Kyrgyzstan, and the Baltic states have undergone a greater degree of market reforms [5]. Growing out-of-pocket fees, both formal and informal, have occurred in many of the health systems, and are thought to have contributed to socioeconomic inequalities in access to health care [6]. However, despite the known weakness of family planning programs in this region, the extent of socioeconomic inequalities in modern contraceptive use in the context of social and economic transition has to our knowledge not been previously studied.

An additional gap in the reproductive health literature both in the region of the Newly Independent States and globally is the inattention to community-level risk factors for modern contraceptive use. Despite a growing recognition of the importance of community characteristics in influencing access to health care, few studies have examined community-level socioeconomic position in association with modern contraceptive use [7-9]. The few that have studied contextual influences on modern contraceptive use are limited to high-income or low-income countries [10-16]. The countries comprising the Newly Independent States are an important setting in which to examine this association, given the magnitude of the public health problem of unmet need for contraceptives and social and economic transformation experienced by the region. The first objective of this analysis was therefore to examine associations of community-level and individual-level socioeconomic status with modern contraceptive use in the region consisting of 10 Newly Independent States (Armenia, Azerbaijan, Moldova, Belarus, Georgia, Kazakhstan, Kyrgyzstan, Tajikistan, Ukraine, and Uzbekistan). The second objective was to examine associations of community-level and individual-level socioeconomic status with modern contraceptive use within each country. Motivating our country-specific analysis was a hypothesis that although the countries of the Newly Independent States have socio-historical similarities, socioeconomic inequalities in modern contraceptive use would vary by country due to unmeasured patterning of factors at country, community-, and individual- levels.

## Methods

### Data sources

The study was a cross sectional analysis of n = 55,204 women aged 15–49 who were married or living with a partner in 10 Newly Independent States. The study used data from the fourth version of the Demographic and Health Surveys (DHS-IV) and the third version of the Multiple Indicator Cluster Surveys (MICS-3), sponsored by the U.S. Agency for International Development and by UNICEF respectively. DHS-IV data was used for Armenia (2005), Azerbaijan (2006), and Moldova, and MICS-3 data was used for Belarus (2005), Georgia (2005), Kazakhstan (2006), Kyrgyzstan (2005–2006), Tajikistan (2005), Ukraine (2005), and Uzbekistan (2006). These countries were chosen based on the availability of data. Although MICS-3 data had been collected in Turkmenistan, this data is not publicly available. Neither DHS-IV nor MICS-3 was conducted in Russia, Latvia, Lithuania, Estonia, and there were no publicly available data comparable to the DHS-IV or MICS-3. Both DHS-IV and MICS-3 utilize a multi-stage sampling design in which household clusters are sampled within each country, and households are then sampled within each cluster. In some countries the surveys utilized an additional sampling stratification at the regional level. Both DHS-IV and MICS-3 consist of in-person interviews. The response rates of the DHS-IV and MICS-3 surveys included in this analysis ranged from 90.3% in Georgia to 99.8% in Belarus and Ukraine [17-26]. The number of women eligible for our analysis ranged from n = 4,156 in Kyrgyzstan to n = 8,855 in Ukraine. Sampling methodology is described in detail in the DHS sampling manual and DHS or MICS country reports [27]. For the survey items relevant to this study, we confirmed through inspection of the English translations of the survey questionnaires that the wording and item choices of the questions were identical or nearly identical for all countries in the sample.

### Study sample

The data used in the analysis are from the individual female respondent data files of the DHS-IV and MICS-3. These data represent responses from individual women aged 15–49 within sampled households to the DHS-IV or MICS-3 Women’s Questionnaire. Although both the DHS-IV and MICS-3 ask whether women are currently married or in a union, the MICS-3 questionnaire does not ask whether respondents have ever been sexually active or are currently sexually active, which makes it impossible to differentiate women who are not currently using contraception because they have not yet started sexual activity from those who are engaged in sexual activity but not using contraception. We therefore only used data from women who reported that they were currently married or in a union, assuming that these women are engaged in current sexual activity. The proportion of women reporting that they were married or in a union ranged from 57% to 71% across the 10 countries in the sample.

### Outcome measure

The outcome in our analysis was current modern contraceptive use (yes/no). The DHS-IV defines modern contraceptives as pills, intrauterine device (IUD), injections, diaphragm, condom, female sterilization, male sterilization, implants, female condom, foam/jelly, lactational amenorrhea, and country-specific modern methods such as emergency contraception. Contraceptive methods included in the survey but not coded as “modern” are periodic abstinence (rhythm method), withdrawal, abstinence, and “other” responses. The DHS-IV survey question was: “*Are you currently doing something or using any method to delay or avoid getting pregnant*? [*If yes*] *Which method are you using*?” The MICS-3 survey question for this variable was: “*Some people use various ways or methods to delay or avoid a pregnancy*. *Are you currently doing something or using any method to delay or avoid getting pregnant*? [*If yes*] *Which method are you using*?” In both surveys, women who reported being currently pregnant were not asked these questions and are therefore excluded from the sample analyzed in our study. However, we were not able to exclude women currently attempting to become pregnant as this information is collected in the DHS-IV but not in the MICS-3. The responses to these questions were recoded into a binary variable for whether or not the respondent currently used any modern method based on the DHS definition of modern methods. If a respondent reported using both a modern and a non-modern method, this was counted as an instance of modern method use.

### Socioeconomic measures

We measured individual socioeconomic status using the respondent’s level of education and household wealth quintile. We recoded the education completion data for each country as a dichotomous variable (higher education vs. less than higher education) due differences in the educational categories in each country regarding vocational schooling, which prevented creation of a more disaggregated categorization (such as primary, secondary, technical, higher). Less than primary, primary, primary vocational, secondary vocational, and secondary were all categorized as secondary education or less, or “Less than higher”. All country surveys included a consistently labeled ‘higher’ education category, so we created a second category “Higher education”. Individual’s household wealth was categorized into quintiles using existing household wealth indices calculated by DHS and MICS for each country dataset [28]. Household wealth quintile is therefore a measure of the household’s relative wealth within the country. Household wealth index is thought to be a good measure of socioeconomic status in countries with a large grey economy and irregular income such as those in this analysis [29,30].

To measure community-level socioeconomic status, we constructed a community wealth variable at the level of the sampling cluster as the percentage of households in the cluster that are in the poorest quintile of the household wealth index aggregated at the national level. To do so we utilized the entire sample of households (as opposed to only married/partnered women in the analytic dataset) in order to minimize same-source bias when examining this variable simultaneously with individual-level household wealth. In defining the variable in this way, we sought to measure the concentration of poverty in each sampling cluster. We considered a linear combination of other variables to define community wealth, such as indoor plumbing and housing material, but there was insufficient community variation in these factors to be considered as proxies of community wealth in this region. The cluster size used in each of the 10 country surveys is fairly consistent. Using the household sampling weights for each data set, the median number of households per cluster across the 10 countries ranged from 8.9 (Kyrgyzstan) to 51 (Ukraine). We assumed that this range of cluster sizes is all within the boundary of what could be reasonably called a ‘community’.

To enhance interpretability, we used a categorical version of the community wealth variable in the analysis. This categorical variable has three possible values: 1 = zero households in the poorest quintile of the household wealth index aggregated at the national level; 2 = greater than zero-25% of households in the poorest quintile; 3 = > 25% of households in the poorest quintile. This categorization was done following inspection of the distribution of percentage of households in the poorest quintile of the household wealth index. We also considered an alternative 4-level categorization (adding a fourth category for communities with more than 50% of the households in the poorest quintile). This comparison was done to ensure that trends in the clusters with the highest concentrations of poverty were not obscured by the 3-level categorization. We found virtually no difference in modern contraceptive use between the 3rd and 4th categories when the 4-level categorization was used, which suggested that information would not be lost by using the more parsimonious 3-level categorization. We therefore adopted the 3-level categorization of the community wealth variable for use in the analysis. In order to ensure that there was an adequate distribution of women from all individual –level wealth quintiles in each community-level wealth tertile, i.e. that the measures were not capturing the same construct, we examined cross tabulations of the two variables by country and found a sufficient sample size in all cells.

### Covariates

We considered all covariates available in both DHS and MICS data in the household and individual female respondent data files. We excluded any variables used in construction of the household wealth indices as these indices were already included among the exposure variables. We included as covariates those variables for which a reasonable theoretical link to modern contraceptive use could be identified based on our review of the literature: woman’s current age, the number of the woman’s children who were alive at the time of the survey, and whether the woman’s household was urban or rural. Both DHS and MICS surveys collect the woman’s age and number of live children by self-report. We categorized woman’s age in 5-year intervals, and we categorized the number of living children after examining the distribution (0, 1, 2, 3, 4 or more). The DHS and MICS data include a variable indicating if the household is urban or rural, which is designated by the survey research team at the time of sampling.

### Statistical analysis

Our first objective was to examine associations of community-level and individual-level socioeconomic status with modern contraceptive use regionally. Therefore, we first constructed a 3-level hierarchical logistic regression model with random intercepts at both the community and country levels in a pooled analysis of all 10 countries. The 3-level model included three units of analysis: countries, communities, and individuals. We chose a random intercept approach to take into account the heterogeneity of baseline risk of MCU at the country- and community-levels, and to account for random variation due to unmeasured country- and community-level variables. Next, we calculated unadjusted odds ratios to estimate associations between each socioeconomic measure with the binary outcome modern contraceptive use. We then calculated adjusted odds ratios to simultaneously adjust for all three socioeconomic variables (education level, household wealth quintile, and community wealth category), age, number of living children, and rural or urban residency.

Our second objective was to examine associations of community-level and individual-level socioeconomic status with modern contraceptive use within each country. To this end we repeated the unadjusted and adjusted models described above for each country using a random intercept at the community level. All models were weighted using sampling weights provided by DHS-IV and MICS-3 administrators. All models were examined for collinearity using the variance inflation factor, but no evidence was found. Multilevel models were estimated using PROC GLIMMIX, SAS v. 9.2.

## Results

### Descriptive statistics

The overall percent of women married/in union using modern contraceptives in the total sample was 41% (Table 1). The lowest level of modern contraceptive use was found in the Caucasus region, with 14% of women in Azerbaijan, 18% in Armenia, and 20% in Georgia using modern contraceptives. In Central Asian countries, Uzbekistan had the highest percent of modern contraceptive users (58%), followed by Kazakhstan (49%), Kyrgyzstan (46%), and Tajikistan (33%). In Eastern Europe, Belarus and Ukraine had the highest percentage of women using modern contraceptives (62% and 59%, respectively), with a lower percentage found in Moldova (45%).

**Table 1:** **Study characteristics by percent using modern contraception among women married or in a union in 10 Newly Independent States **(**n** = **55**,**204**)

** Characteristic**	** n**	**All Countries n** **=** **55,204**	**ARM n** **=** **4,170**	**AZE n** **=** **5,260**	**BEL n** **=** **4,172**	**GEO n** **=** **6,183**	**KAZ n** **=** **8,369**	**KYR n** **=** **4,156**	**MOL n** **=** **4**,**892**	**TAJ n** **=** **6**,**007**	**UKR n** **=** **4,169**	**UZB n** **=** **8,855**
		**%**	**%**	**%**	**%**	**%**	**%**	**%**	**%**	**%**	**%**	**%**
**Total**	55, 204	41	18	14	62	20	49	46	45	33	59	58
**Community wealth category**		(***)	(***)	(***)	(**)	(***)	(***)	(*)	(***)	(***)	(***)	
Poorest	17,672	35	15	11	54	15	39	48	40	28	46	60
Middle	15,542	41	20	13	55	20	50	43	42	31	59	59
Richest	21,991	44	22	18	59	25	55	45	49	39	63	59
**Household wealth quintile**		(***)	(***)	(***)	(***)	(***)	(***)	(***)	(***)	(***)	(***)	(*)
Poorest	10,070	35	12	11	49	12	40	47	37	26	43	61
Poorer	10,747	38	16	11	53	16	45	40	39	32	53	60
Middle	11,355	40	17	10	59	18	49	43	43	32	58	61
Richer	11,334	43	22	18	60	23	51	48	46	36	66	57
Richest	11,699	46	29	21	59	28	57	48	51	39	67	57
**Education**		(***)	(***)	(***)	(***)	(***)	(*)		(***)	(***)	(**)	
Less than higher	44,060	39	17	13	55	17	48	45	42	32	56	60
Higher	11,144	46	28	25	63	27	51	48	51	46	61	57
**Age**		(***)	(***)	(***)	(***)	(***)	(***)	(***)	(***)	(***)	(***)	(***)
15-19 years	1,146	16	4	3	51	9	28	15	34	4	40	19
20-24 years	7,189	31	19	9	60	22	38	29	41	16	45	40
25-29 years	9,380	45	27	20	64	26	51	49	51	32	65	61
30-34 years	9,262	49	27	21	64	27	59	54	53	42	67	67
35-39 years	9,252	49	22	18	69	23	57	61	53	46	67	72
40-44 years	10,009	42	18	12	57	17	53	53	45	36	63	69
45-49 years	8,967	27	7	7	34	9	32	26	25	29	43	47
**Number of living children**		(***)	(***)	(***)	(***)	(***)	(***)	(***)	(***)	(***)	(***)	(***)
0	4,706	11	2	1	26	3	11	10	22	1	34	4
1	11,466	41	20	13	61	23	47	34	45	11	66	42
2	19,478	44	23	19	59	22	59	50	50	36	61	66
3	10,660	43	18	14	50	18	50	58	42	41	52	73
4 or more	8,895	46	12	14	54	11	43	49	39	40	27	67
**Residence type**		(***)	(***)	(***)	(***)	(***)	(***)		(***)	(***)	(***)	(**)
Rural	29,111	39	16	10	52	16	44	45	41	31	50	60
Urban	26,093	43	21	18	59	24	52	47	48	38	62	57

P-value from chi-squared test of association: * = p < 0.05; ** = p < 0.01; *** = p < 0.001.

### Pooled regional analyses

In the unadjusted pooled model examining 10 countries simultaneously with random intercepts at cluster and country levels, community socioeconomic status was associated with modern contraceptive use, such that women living in the poorest communities had 0.69 times the odds of using modern contraceptives relative to those in the richest (95% Confidence Interval (CI) = 0.66, 0.73), and women living in the middle-level communities had 0.83 times the odds of using modern contraceptives (95% CI = 0.79, 0.87) (Table 2). There was an inverse association between individual socioeconomic status and modern contraceptive use; women in the poorest quintile of household wealth had an odds of contraceptive use 0.62 times that of those in the richest quintile (95% CI = 0.58, 0.66), with odds ratios in the poorer and middle quintiles of 0.69 (95%CI = 0.65, 0.73), and 0.76 (95%CI = 0.72, 0.81), respectively. Women with a lower level of education also had a decreased odds of modern contraceptive use (OR = 0.75, 95%CI = 0.71, 0.78). In the pooled model adjusting for covariates and mutually adjusting for other socioeconomic measures, odds ratios showed similar trends but were of somewhat smaller magnitude (Table 2).

**Table 2:** **Regional unadjusted and adjusted odds ratios for community and individual socioeconomic status and modern contraception use**, **women married or in a union in 10 Newly Independent States **(**N** = **55**,**204**)

**Socioeconomic measures**	**Unadjusted odds ratio95%Confidence interval**	**Adjusted**^**1**^**odds ratio95%Confidence interval**
**Community wealth category**		
Poorest	0.69 (0.66–0.73)	0.82 (0.76, 0.89)
Middle	0.83 (0.79–0.87)	0.93 (0.87–0.99)
Richest	1.00	1.00
**Household wealth quintile**		
Poorest	0.62 (0.58–0.66)	0.68 (0.62–0.75)
Poorer	0.69 (0.65–0.73)	0.75 (0.69–0.81)
Middle	0.76 (0.72–0.81)	0.82 (0.76–0.88)
Richer	0.87 (0.83–0.93)	0.93 (0.87–0.99)
Richest	1.00	1.00
**Education**		
Less than higher	0.75 (0.71–0.78)	0.75 (0.71–0.79)
Higher	1.00	1.00

^1^Adjusted for age, number of living children, residence type, community wealth, household wealth, and education.

### Country-specific analyses

In country-specific unadjusted models, women living in the poorest communities had a lower odds of modern contraceptive use than those in the richest communities in all countries except for Belarus, Kyrgyzstan, and Uzbekistan, with odds ratios ranging from 0.48 in Ukraine (95%CI = 0.32, 0.73) to 0.69 in Armenia (95%CI = 0.54, 0.88) (Table 3). The odds ratio for the poorest communities relative to the richest in Belarus was 0.82, (95%CI = 0.64, 1.04), in Kyrgyzstan it was 1.20 (95% CI = 0.96, 1.48 and in Uzbekistan it was 1.03 (95%CI = 0.87, 1.21).

**Table 3:** **Unadjusted odds ratios for community and individual socioeconomic status and modern contraception use**, **women married or in a union in 10 Newly Independent States **(**n** = **55**,**204**)

**Socioeconomic measures**	**ARM95%CI**	**AZE95%CI**	**BEL95%CI**	**GEO95%CI**	**KAZ95%CI**	**KYR95%CI**	**MOL95%CI**	**TAJ95%CI**	**UKR95%CI**	**UZB95%CI**
**Community wealth category**										
Poorest	0.69 (0.54, 0.88)	0.58 (0.45, 0.74)	0.82 (0.64, 1.04)	0.49 (0.39, 0.62)	0.57 (0.48, 0.67)	1.20 (0.96, 1.48)	0.68 (0.58, 0.80)	0.59 (0.48, 0.72)	0.48 (0.32, 0.73)	1.03 (0.87, 1.21)
Middle	0.92 (0.71, 1.20)	0.65 (0.50, 0.84)	0.85 (0.66, 1.10)	0.73 (0.56, 0.95)	0.84 (0.72, 0.98)	1.08 (0.85, 1.37)	0.75 (0.64, 0.88)	0.72 (0.60, 0.87)	0.80 (0.38, 1.64)	1.03 (0.89, 1.19)
Richest	1.00	1.00	1.00	1.00	1.00	1.00	1.00	1.00	1.00	1.00
**Household wealth quintile**										
Poorest	0.36 (0.27, 0.48)	0.47 (0.35, 0.62)	0.68 (0.52, 0.89)	0.33 (0.25, 0.44)	0.59 (0.49, 0.70)	0.96 (0.75, 1.22)	0.54 (0.45, 0.67)	0.57 (0.46, 0.70)	0.47 (0.32, 0.69)	1.27 (1.08, 1.49)
Poorer	0.45 (0.35, 0.59)	0.46 (0.35, 0.61)	0.78 (0.61, 0.99)	0.49 (0.38, 0.63)	0.64 (0.54, 0.76)	0.72 (0.57, 0.91)	0.58 (0.48, 0.71)	0.77 (0.63, 0.93)	0.55 (0.39, 0.78)	1.16 (1.00, 1.35)
Middle	0.52 (0.40, 0.67)	0.44 (0.33, 0.57)	0.92 (0.73, 1.16)	0.52 (0.40, 0.66)	0.70 (0.59, 0.82)	0.88 (0.70, 1.11)	0.71 (0.59, 0.85)	0.73 (0.60, 0.88)	0.69 (0.53, 0.90)	1.17 (1.0, 1.36)
Richer	0.70 (0.56, 0.89)	0.85 (0.67, 1.07)	1.02 (0.82, 1.27)	0.81 (0.65, 0.99)	0.80 (0.69, 0.94)	1.03 (0.82, 1.28)	0.82 (0.68, 0.97)	0.85 (0.71, 1.02)	0.99 (0.77, 1.28)	1.00 (0.87, 1.17)
Richest	1.00	1.00	1.00	1.00	1.00	1.00	1.00	1.00	1.00	1.00
**Education**										
Less than higher	0.55 (0.46, 0.67)	0.49 (0.39, 0.60)	0.72 (0.60, 0.85)	0.61 (0.53, 0.71)	0.91 (0.82, 1.02)	0.90 (0.77, 1.06)	0.72 (0.62, 0.83)	0.62 (0.50, 0.78)	0.89 (0.76, 1.04)	1.11 (0.95, 1.29)
Higher	1.00	1.00	1.00	1.00	1.00	1.00	1.00	1.00	1.00	1.00

With regard to individual-level socioeconomic status, women in the poorest quintile of household wealth had lower odds of modern contraceptive use in all countries except Kyrgyzstan and Uzbekistan, with odds ratios ranging from 0.33 in Georgia (95%CI = 0.25, 0.44) to 0.68 in Belarus (95%CI = 0.52. 0.89) (Table 3). In these countries there was a trend of increasing modern contraceptive use with increasing wealth. In Kyrgyzstan those in the poorest quintile of household wealth had similar odds of modern contraceptive use as those in the richest quintile (OR = 0.96, 95%CI = 0.75, 1.22) and there was no clear trend of increasing contraceptive use with increasing wealth. In Uzbekistan, those in the poorest quintile of household wealth had an increased odds of modern contraceptive use relative to those in the richest (OR = 1.27, 95%CI = 1.08, 1.49), as did those in the poorer and middle quintiles (OR = 1.16, 95%CI = 1.00, 1.35; OR = 1.17, 95%CI = 1.00, 1.36). The findings regarding our second measure of individual-level socioeconomic status, education, diverged slightly from the findings regarding individual wealth. Women with lower education had a reduced odds of modern contraceptive use in most countries, with odds ratios ranging from 0.49 (95%CI = 0.39, 0.60) in Azerbaijan to 0.62 in Tajikistan (95%CI = 0.50, 0.78). The exceptions were Kazakhstan, Kyrgyzstan, Ukraine, and Uzbekistan, where there was no association.

In adjusted country-specific analyses that mutually controlled for all socioeconomic measures, as well as age, number of living children, and urban or rural residence, estimates for community wealth were notably different. In Armenia, Azerbaijan, Belarus, Georgia, Moldova, and Ukraine, there was no longer a statistically significant association between living in the poorest community and modern contraceptive use (Table 4). In Kazakhstan, Tajikistan, and Uzbekistan, however, there remained after adjustment for individual socioeconomic status and other covariates a statistically significant reduced odds of modern contraceptive use among those in the poorest communities relative to those in the richest. (Adjusted odds ratio (aOR) for Kazakhstan = 0.61, 95%CI = 0.46, 0.79; aOR for Tajikistan = 0.73, 95%CI = 0.54, 0.99; aOR for Uzbekistan = 0.75, 95%CI = 0.58, 0.97). In Kyrgyzstan, there was an increased odds of modern contraceptive use among the poorest communities (aOR for poorest tertile = 1.68, 95%CI = 1.22, 2.31; aOR for middle tertile = 1.33, 95%CI = 1.01, 1.75).

**Table 4:** Adjusted* **odds ratios for community and individual socioeconomic status and modern contraception use**, **women married or in a union in 10 Newly Independent States **(**n** = **55**,**204**)

**Socioeconomic measures**	**ARM95%CI**	**AZE95%CI**	**BEL95%CI**	**GEO95%CI**	**KAZ95%CI**	**KYR95%CI**	**MOL95%CI**	**TAJ95%CI**	**UKR95%CI**	**UZB95%CI**
**Community wealth category**										
Poorest	1.19 (0.77, 1.86)	1.52 (0.96, 2.41)	1.23 (0.84, 1.81)	0.92 (0.59, 1.45)	0.61 (0.46, 0.79)	1.68 (1.22, 2.31)	0.88 (0.64, 1.20)	0.73 (0.54, 0.99)	0.33 (0.10, 1.04)	0.75 (0.58, 0.97)
Middle	1.19 (0.89, 1.59)	1.19 (0.85, 1.65)	1.02 (0.76, 1.36)	1.13 (0.79, 1.62)	0.90 (0.73, 1.11)	1.33 (1.01, 1.75)	0.88 (0.68, 1.13)	0.81 (0.63, 1.04)	0.59 (0.17, 2.08)	0.86 (0.70, 1.05)
Richest	1.00	1.00	1.00	1.00	1.00	1.00	1.00	1.00	1.00	1.00
**Household wealth quintile**										
Poorest	0.35 (0.24, 0.52)	0.52 (0.35, 0.78)	0.76 (0.53, 1.07)	0.39 (0.26, 0.85)	0.74 (0.58, 0.96)	0.64 (0.46, 0.90)	0.59 (0.43, 0.82)	0.61 (0.46, 0.80)	0.65 (0.37, 1.15)	1.14 (0.91, 1.43)
Poorer	0.46 (0.33, 0.63)	0.56 (0.39, 0.80)	0.81 (0.60, 1.09)	0.57 (0.39, 0.85)	0.75 (0.60, 0.94)	0.52 (0.38, 0.70)	0.61 (0.46, 0.83)	0.84 (0.65, 1.08)	0.58 (0.36, 0.91)	1.13 (0.92, 1.38)
Middle	0.53 (0.41, 0.70)	0.50 (0.36, 0.70)	1.00 (0.78, 1.30)	0.57 (0.40, 0.81)	0.77 (0.62, 0.94)	0.74 (0.56, 0.98)	0.74 (0.56, 0.97)	0.89 (0.70, 1.13)	0.60 (0.45, 0.80)	1.15 (0.95, 1.39)
Richer	0.74 (0.58, 0.94)	0.94 (0.73, 1.20)	1.04 (0.82, 1.31)	0.89 (0.71, 1.12)	0.88 (0.74, 1.04)	0.91 (0.71, 1.17)	0.83 (0.67, 1.03)	0.97 (0.78, 1.22)	0.92 (0.71, 1.21)	1.03 (0.86, 1.23)
Richest	1.00	1.00	1.00	1.00	1.00	1.00				
**Education**										
Less than higher	0.64 (0.52, 0.78)	0.55 (0.44, 0.70)	0.65 (0.55, 0.78)	0.67 (0.57, 0.78)	0.88 (0.78, 0.99)	0.80 (0.67, 0.96)	0.73 (0.62, 0.86)	0.64 (0.50, 0.81)	0.94 (0.79, 1.11)	1.18 (1.00, 1.40)
Higher	1.00	1.00	1.00	1.00	1.00	1.00	1.00	1.00	1.00	1.00

*Adjusted for age, number of living children, residence type, community wealth, household wealth, and education.

After adjustment for covariates, associations between individual-level socioeconomic status and modern contraceptive use were slightly reduced in magnitude for most countries, and estimates for Belarus, Ukraine, and Uzbekistan were no longer statistically significant (Table 4). Kyrgyzstan was the exception – after adjustment the estimate of the association between individual-level socioeconomic status was reversed, with those in the lower three quintiles of household wealth at a reduced odds of modern contraceptive use compared to those in the richest (aOR for poorest quintile = 0.64, 95%CI = 0.46, 0.90). After adjustment, estimates for education for most countries strengthened slightly in magnitude, and the only two countries for which lower education was not associated with a decreased odds of modern contraceptive use were Ukraine and Uzbekistan.

## Discussion

Our study found striking evidence of socioeconomic inequalities in modern contraceptive use in countries of the Newly Independent States. We found both community-level and individual-level socioeconomic inequalities in modern contraceptive use at a regional level. However, country-level analyses revealed a more mixed picture. In 4 of 10 countries community-level socioeconomic status was significantly associated with modern contraceptive use. We also found associations in 7 of 10 countries for individual-level socioeconomic status and in all but two countries for education. In addition, some sub-regional similarities emerged in Central Asia and in Caucasus, but not in Eastern Europe.

Our study contributes to a scant literature on community-level socioeconomic status and modern contraceptive use. The few studies that studied community-level socioeconomic factors and modern contraceptive use had mixed results [10-16]. The study that most closely resembled ours examined community-level mean household assets using Demographic Health Survey data in association with modern contraceptive use in 6 countries in Africa and found an association of increased community-level assets with increased modern contraceptive use only in one of the six countries, Burkina Faso [15]. The authors suggested this finding might be due to increased economic development in Burkina Faso. In the three studies on community-level socioeconomic characteristics and contraceptive use in high-income countries, an inverse association was found between levels of community disadvantage and contraceptive use [10-12]. Our results provided limited support to the hypothesis that community-level disadvantage is associated with lower contraceptive use primarily in the context of a high level of economic development. In Central Asia the strongest association between poor community-level socioeconomic status and modern contraceptive use was found in the most developed country, Kazakhstan, and in Eastern Europe the same could be said regarding Ukraine, although in Ukraine the association was not statistically significant. Further research might examine more closely associations between community-level inequalities and national economic development levels.

### Central Asia

Several similarities emerged in Central Asian countries. First, the only countries in our study in which community-level socioeconomic status had a statistically significant association with modern contraceptive use after adjustment for individual-level factors were Central Asian countries. In three of these countries, Kazakhstan, Tajikistan, and Uzbekistan, women in the poorest communities were less likely to use modern contraceptives, while in Kyrgyzstan, women living in the poorest communities were *more* likely to use modern contraceptives. Central Asian countries also were similar in that individual-level socioeconomic status was positively associated with modern contraceptive use in all countries except Uzbekistan, in which there were no individual-level differences in use of modern contraceptives in unadjusted or adjusted analyses. In addition, Uzbekistan is the only country in our analysis in which the odds of modern contraceptive use were higher among women with lower education, and also had the highest overall percent use in Central Asia (58%). Previous literature on modern contraceptive in Central Asia, although scant, may elucidate our findings.

One study previously examined modern contraceptive use in Uzbekistan. After Uzbekistan gained independence, the government launched a new family planning program in an effort to reduce the use of abortion as contraception. This program centered on use of the IUD, and has been attributed to increasing the use of modern contraceptives in Uzbekistan [31]. If the government family planning program targeted less educated women, it could explain our findings. This apparent equality in Uzbekistan might be considered a success of a targeted government-sponsored family planning program, and is consistent with research in other middle-income countries showing that higher provision of public capital may compensate for low levels of human capital, i.e. education, in regards to modern contraceptive use [32]. On the other hand, the government reproductive health program in Uzbekistan has been criticized for being coercive and constraining reproductive choice [33]. The potential of a trade-off between reproductive choice and equity in modern contraceptive use is important to consider in efforts to achieve reproductive health equity.

Our findings also support a previous study comparing Kazakhstan and Belarus that found greater inequality in modern contraceptive use in Kazakhstan than in Belarus [34]. The authors hypothesized that a reason for the higher degree in equality in Belarus was due to the fact that the health system in Kazakhstan had undergone more market-based reforms than that in Belarus. Our findings regarding both community and individual wealth support this hypothesis – in adjusted analyses, individual wealth or community wealth were not associated with modern contraceptive use in Belarus, whereas in Kazakhstan women in the poorest communities and in the poorest quintiles of individual wealth were less likely to use modern contraceptives.

### Caucasus

We found the most marked inequalities in modern contraceptive use by individual socioeconomic status in the Caucasus region, including Georgia, Armenia, and Azerbaijan, although community wealth was not independently associated with modern contraceptive use in these countries. These countries also had the lowest overall rates of modern contraceptive use. Our findings build on previous research regarding individual-level inequalities in access to health care and the need for family planning in this region.

Previous research identified the Caucasus region as a region with large inequalities in access to health care. A study of 8 countries of the former Soviet Union (Armenia, Belarus, Georgia, Kazakhstan, Kyrgyzstan, Moldova, Russia, and Ukraine) in 2001 found inequalities in access to health care in all countries, but that the inequalities were most marked in Georgia and Armenia, and that these inequalities persisted in a 2010 follow up survey [35,36]. Our study builds on this research by showing that inequalities by individual socioeconomic status extend to access to contraceptives in these countries.

Previous research has also identified an urgent need for increased family planning programs in the Caucasus. In Georgia, a comparison study between 2005 and 2010 suggested that despite having declined by 16% (from 3.7 to 3.1 abortions per woman), abortion rates were still the highest documented worldwide. In Azerbaijan and Armenia, where abortion rates are also high, there is also a clear need for family planning programs. A survey conducted in 2005 in Azerbaijan found that among married women who have never used modern contraceptives, the most frequent reasons stated were fear of side effects (35.8%) and lack of knowledge about family planning (31.8%) [37]. The same study found lack of availability of contraceptives at health centers, particularly outside the capital city. A survey in Armenia in 1995 of women attending an abortion clinic found that the most frequent reason for not using contraception was lack of knowledge (60%), followed by unavailability or cost (15%), and that most women (90%) wanted further information on contraception [38]. Another study in Armenia in 2002 found that only 19.4% of post-partum care providers provided information to patients regarding birth spacing and contraceptives [39]. These previous findings suggest that lack of knowledge may be an important factor contributing to the individual-level inequities in modern contraceptive use in the region, and that community-based programs to increase knowledge of family planning are needed. An example of such a program can be found in Armenia, where a national family planning media campaign in 2000 successfully increased contraceptive use. Considering the inequalities identified in Armenia in this analysis, it would be of interest for future interventions to evaluate whether they are effective across communities and individuals of varying socioeconomic status [40].

### Eastern Europe

We found few commonalities between the Eastern European Newly Independent States that we studied: Belarus, Moldova, and Ukraine. These countries share cultural and religious characteristics, but differ in levels of democracy and development, with Belarus having the most totalitarian government in Eastern Europe, and Moldova being the poorest Eastern European country. The general fertility pattern in Eastern Europe relative to Western Europe tends to be earlier childbearing followed by abortion or contraception to space or limit subsequent children [41]. Despite these similarities, there are few similarities in patterns of inequalities between countries. Likewise, the countries in the Caucasus region displayed more similar patterns of modern contraceptive use, despite large cultural and religious differences between countries. These findings suggest that from a regional perspective, cultural and religious differences do not drive inequalities in modern contraceptive use.

### Future research

This analysis describes patterns in individual-level and community-level inequalities in modern contraceptive use that generate hypotheses regarding multilevel factors influencing contraceptive use. Three factors that merit future research in the context of the post-Soviet transition are gender equality, social capital, and health system characteristics. Despite official policies proclaiming gender equality during the Soviet era, power differentials between men and women differed historically throughout the region [2,42]. Eastern European countries of the Soviet Union (Belorussia, Moldova, and Ukraine) shared relatively more egalitarian gender beliefs and women had greater access to education and employment [43]. In contrast, countries of the Caucasus and Central Asia observed more traditional values and cultural norms in respect to women and their roles in the family [44-47]. Future research might explore the role of evolving male/female power dynamics in socioeconomic inequalities at the individual- and community-level.

The influence of social capital on modern contraceptive use is another important avenue for future research to unpack the community-level and individual-level inequalities described in this analysis. Social capital is a known determinant of access to health care in the former Soviet Union [48]. Social capital in the form of both personal networks and communication networks are also known to be influential to contraceptive use in Eastern Europe and elsewhere via access to resources and knowledge [49]. The Newly Independent States are an opportune region to explore associations between social capital and inequalities in modern contraceptive use due to their shared experience of economic transition.

Finally, health system characteristics themselves are an important macro-level determinant of equality in access to health care [50]. Because the ten countries included in our analysis have undergone health system reform to varying degrees since the dissolution of the Soviet Union, they present an ideal opportunity to measure the impact of these reforms on family planning. Future researchers might collect data on specific characteristics of the health system of each country, such as degree of privatization and introduction of user fees, including the extent to which they apply to family planning programs, and conduct in-depth analysis of the association of these characteristics with trends in inequalities in modern contraceptive use.

### Strengths and limitations

Our study had several strengths. First, ours is one of the few studies to consider how community-level socioeconomic status influences modern contraceptive use, and how these associations differ across countries. To do so, we were one of very few studies that have created a community-level socioeconomic measure using MICS or DHS data, an approach that should be considered more frequently. Second, we drew on the shared sociopolitical history of the Newly Independent States to examine both regional and within country associations between both community- and individual-level socioeconomic status and modern contraceptive use. This novel approach serves to stimulate hypotheses regarding within country and between country differences in reproductive health and family planning services. Finally, we took advantage of the comparability and comprehensiveness of MICS and DHS survey data on contraceptive use to create a unique dataset encompassing ten Newly Independent States.

Despite the strengths of our analyses, there are several limitations to be noted. First, we constructed our measure of community-level socioeconomic status by aggregating an individual-level measure, and thus may not have fully distinguished the poverty level of the community from the individuals who comprise it. In order to minimize this bias, we used the entire sample from the surveys to compose this measure, as opposed to the sample of married/partnered women for analysis. Second, we examined cross tabulations between community and individual measures to ensure that there were not empty strata, such that although the measures were correlated, there were poor individuals in all levels of community socioeconomic status and vice versa. Another limitation regarding the measure of community wealth is that it is not strictly comparable among countries. We adopted the approach of using within-country wealth indexes so that a community would be labeled ‘poor’ relative to its own country; however, this means that a poor community in Ukraine is not necessarily at the same absolute level of poverty as a community in Uzbekistan. This may limit inferences for total associations between community-level wealth and modern contraceptive use.

There also are limitations in the measures of household wealth. The distribution of wealth index scores varied by country; some countries had larger absolute difference in wealth when comparing poorest and richest quintiles than in other countries. Such differences in absolute wealth disparity between poorest and richest quintile may influence the comparative magnitude of the coefficients for quintiles of wealth across countries, as well as the degree to which we see a trend within each country of increasing contraceptive use with increasing wealth. Therefore, interpretation of comparisons and trends between countries should be cautious.

The measure of educational status also has some limitations. Women in the youngest age category, aged 15–19, would not have had an opportunity to complete higher education. Because these women have a lower prevalence of modern contraceptive use, they may have biased the estimate for the association between low education and modern contraceptive use away from the null. However, the number of women in this age category is limited (n = 1146), representing only 2% of the study sample. Additionally, we may have not fully captured all relevant aspects of educational status since we did not include partner’s level of education. Understanding better how the socioeconomic status of the woman-partner dyad influences modern contraceptive use is fertile ground future research.

Another limitation is that because we did not have information on sexual activity and therefore limited analyses only to women who were married or in a union, our results are not generalizable to the all women in NIS countries. In particular, because younger women may be less likely to be married or in a union, inference from our findings for younger women should be made with care. We also did not have information on pregnancy intention for the seven countries for which we used MICS-3 data. Because of this we did not take pregnancy intention into account in our analysis, and were therefore unable to measure “unmet need” for contraception.

## Conclusions

Our study examined socioeconomic inequalities in modern contraceptive use in middle-income countries with health care systems in transition, characterized by historical lack of family-planning programs, growing out-of-pocket fees, varying degrees of decentralization, and differing funding sources. Community-level socioeconomic status had the greatest influence in Central Asian countries, with women from poor communities being less likely to use modern contraceptives. Individual-level inequalities in which women from poor households were least likely to use modern contraceptives were most pronounced in the Caucasus, where the lowest prevalence of modern contraceptive use was found. We also identified inequalities in Eastern European countries, but with no specific pattern. These findings demonstrate that modern contraceptive use is a complex issue that is likely to have multilevel determinants and stimulate hypotheses about which contextual factors may influence reproductive health inequalities. As reproductive health and family planning programs are strengthened in the Newly Independent States in the context of ongoing health system reform, policy makers should monitor inequalities to ensure that all communities and individuals have equal access to modern contraceptives.

## Competing interests

The authors declare that they have no competing interests.

## Authors’ contributions

TJ conceived of the study and drafted the Introduction, Results, Discussion, and Conclusions. SP carried out the analyses and designed the tables. LI made substantial contributions to the conception and design and interpretation of data. EB revised the manuscript critically for important intellectual content. All authors read and approved the final manuscripts.
